# Batf3-cDC1 control Th1 and fungicidal responses during cryptococcal meningitis: is this enough to control meningitis?

**DOI:** 10.1128/mbio.00375-24

**Published:** 2024-09-10

**Authors:** Carolina Coelho

**Affiliations:** 1 MRC Centre for Medical Mycology at University of Exeter, University of Exeter, Exeter, United Kingdom; Albert Einstein College of Medicine, Bronx, New York, USA

**Keywords:** dendritic cells, cDC1, cDC2, Batf3, *Cryptococcus*, fungal, medical mycology

## Abstract

Dendritic cells are crucial for bridging innate and adaptive immunity. Cryptococcosis, caused by *Cryptococcus neoformans* and *Cryptococcus gattii*, is responsible for >15% of AIDS-related deaths. A recent study by Xu et al. showed that *Batf3*-dependent conventional type 1 dendritic (cDC1) cells are key players in generating IFNγ^+^ CD4^+^ T cell and fungicidal lung and brain tissue-resident responses during murine cryptococcosis, contributing to fungal clearance in the lungs and brain of mice (J. Xu, R. Hissong, R. Bareis, A. Creech, et al., mBio 15:e02853-23, 2024, https://doi.org/10.1128/mbio.02853-23). However, despite their critical role, the depletion of *Batf3*-dependent cDC1 cells did not significantly alter overall mouse survival or disease progression, highlighting the complex immune regulation required to survive cryptococcal infection and the need for further research in medical mycology.

## COMMENTARY

Dendritic cells (DCs) are sentinel leukocytes that bridge innate and adaptive immunity (1). Conventional DCs (cDCs) develop from a lineage of hematopoietic stem cells, in a fms-like tyrosine kinase 3 (*Flt3*)*-*dependent pathway. At the pre-cDC stage, they egress from the bone marrow to colonize lymphoid and nonlymphoid organs, where they differentiate into resting cDCs that function as sentinel cells of tissues (1). From the pre-cDC stage, they differentiate into two subsets: cDC1 differentiation depends on *Irf8*, *Batf3*, *Nfil3*, and *Id2*, and cDC2 relies on *Irf4*, *Irf2,* and *Traf6*. cDC1s account for approximately 30% of total cDCs in tissues and 40% in lymphoid organs, with cDC2s comprising the rest of the population. These subsets are found to differ based on location within the mucosa and epithelium of each organ and in their relative expression of sensing receptors pathogen-associated molecular patterns and danger-associated molecular patterns. These differences, albeit subtle, are thought to impact the contribution of each cDC subset to effective immunity. This is further complicated by functional redundancy: other subsets, such as monocyte-derived cells, can acquire markers and functions of dendritic cells (usually abbreviated moDC) (2). The specific role of each subset is an area of active research.

Cryptococcosis is a fungal disease caused by *Cryptococcus neoformans* and *Cryptococcus gattii*. The global death toll from cryptococcosis remains significant, especially in regions with high rates of HIV/AIDS and limited access to healthcare. During cryptococcal challenge, the depletion of DC and macrophages subsets with a CD11c-diphtheria toxin receptor model reduced mouse survival (3), and others showed that dendritic cells are capable of killing cryptococci and presenting cryptococcal antigen (4, 5). For cDCs, the killing of fungi was via lysosomal components, including cathepsin B (6), and for pDCs, killing was dependent on reactive oxygen species (ROS) and dectin-3 (7). In the specific case of *C. neoformans*, an accumulation of immature DC in the lungs and lung-associated lymph nodes accompanied by *Ym1*^+^ macrophages prevents protective adaptive responses and seems to contribute to death in mouse models (8). Interestingly, these responses were decreased in the hypovirulent strains deleted for fungal urease, strains that show decreased growth at neutral pH and increased growth inside macrophage phagosome (9). The induction of protective DC responses required TNFα (10) for the epigenetic stabilization of an effective anticryptococcal response in the lungs (11).

Knowledge of the role of each cDC subset during fungal infections is still limited. We can now use the basic leucine zipper ATF-like transcription factor 3 *Batf3^−/−^* mouse as a model to selectively deplete cDC1, relative to other DC subsets. There is a strong rationale for cDC1 playing important roles in antifungal immunity as IL-12 is a key cytokine for antifungal immunity (12), and cDC1 cells were found to produce high levels of IL-12 (13, 14) during *Toxoplasma gondii* parasite infections. Thus far, cDC1 were shown to be major producers of type I interferon in the lungs of mice infected with *Histoplasma capsulatum* (15, 16). In dermal candidiasis, the use of this *Batf3^−/−^* model showed that cDC1 play a dispensable role but may instead be important for certain steps of immunity against systemic candidiasis (16, 17).

In this recent study by Xu et al. (18), the essential role of *Batf3*-dependent cDC1s in the lung and brain anticryptococcal immunity was demonstrated. Ablation of *Batf3*-cDC1 impacted the development of Th1 and fungicidal responses in the brain and lung, albeit with little impact on mouse survival. The authors first used single-cell RNA sequencing to identify that *Batf3*-dependent cDC1 exhibited a unique mRNA signature during murine cryptococcosis, strongly upregulating transcripts related to T cell recruitment and Th1 polarization, such as *Il12b*, *Stat4*, and *Ccl22*. This study further demonstrated an essential role for *Batf3^−/−^*cDC1 in generating protective T cell responses, ascertained via reduced immune infiltrates in the lung and brain, including IFNγ-producing CD4^+^ T cells and CD8^+^ T cells, and reduced type 1 cytokine production. At late stages of infection, *Batf3* deletion leads to 100-fold and 10-fold increased fungal burden in the lung and brain, respectively.

Notably, the removal of *Batf3*-dependent cDC1s led to a reduced *Nos2* expression in tissues and reduced iNOS protein expression in brain microglia and inflammatory monocytes, indicating that cDC1 activity culminates in the activation of phagocytes required for fungal clearance. Since CD4 T cell depletion experiments did not exacerbate fungal burden in *Batf3*^−/−^ mice, there is a requirement for cDC1 activation of T cells in fungal clearance.

We note that *Batf3-*dependent effects are attributed to the depletion of cDC1 cells. However, *Batf3* expression may regulate other cells, such as cDC2, CD8^+^ T cells, and tissue-resident cells, which express low levels of *Batf3*. In particular, there is a significant expression of *Batf3* in microglia during infection and LPS challenge (19), and thus, it is possible that *Batf3* may be important in regulating the activity of these brain-resident macrophage populations. These confounders will need to be ruled out in future studies.

Important conundrums remain regarding the role of cDC2 and monocyte-derived DC subsets in experimental cryptococcosis. In previous work, CD11c-Cre-IRF4^fl/fl^ mice were used to deplete the cDC2 subset; in this model, cDC2 in lungs were shown to induce a Th2-type polarization in the lungs (20) (brain responses were not studied in this publication), a profile associated with nonprotective responses in cryptococcosis. Taken together with the work of Xu et al. (18), these results provide evidence that cDC1 likely play a predominant role compared to other DC subsets in augmenting the Th1 response in both lungs and brains of mice.

Finally, despite all these positive antifungal responses, mouse survival was unchanged by *Batf3* deletion. The authors previously showed that mouse mortality in this model of intravenous infection with 52D strain was dependent on CD4^+^ T cell and CCR2-monocyte infiltration and subsequent immunopathology, and independent of fungal burden in brains (21, 22). However, *Batf3*^−/−^ mice CD4^+^ T cell infiltration was greatly reduced in the brain and lungs, which suggests an alternative route of immunopathology and mortality. As discussed by the authors, there is also a potential role for CCR2-monocytes, and potentially their finer polarization status, in driving pathology (22), which may be investigated in the future. Additionally, this puzzling result should be confirmed in other cryptococcal models, including pulmonary infection models, to ensure this is not a peculiarity of the experimental model, which is sensitive to variables such as pathogen strain and dose. Still, this result underscores a conundrum that is striking in cryptococcal meningitis and associated immune reconstitution inflammatory syndrome: fungal burden, and decrease in fungal burden is not sufficient to predict the severity of disease, through mechanisms not fully understood of immune deregulation and tissue damage (23). These make the clinical management of cryptococcosis in HIV+ and non-HIV patients very difficult.

This is one of the first studies to examine the role of DC and their subsets in controlling fungicidal and protective immune responses against *C. neoformans*. Continuation of research efforts is crucial for gaining a comprehensive understanding of these processes, which will be instrumental in informing the development of much-needed host-directed therapies to treat lethal cryptococcosis.
